# Neurosteroids in Pain Management: A New Perspective on an Old Player

**DOI:** 10.3389/fphar.2018.01127

**Published:** 2018-10-02

**Authors:** Sonja L. Joksimovic, Douglas F. Covey, Vesna Jevtovic-Todorovic, Slobodan M. Todorovic

**Affiliations:** ^1^Department of Anesthesiology, University of Colorado Denver, Anschutz Medical Campus, Aurora, CO, United States; ^2^Department of Developmental Biology, School of Medicine, Washington University in St. Louis, St. Louis, MO, United States; ^3^Taylor Family Institute for Innovative Psychiatric Research, School of Medicine, Washington University in St. Louis, St. Louis, MO, United States; ^4^Neuroscience Graduate Program, University of Colorado Denver, Anschutz Medical Campus, Aurora, CO, United States

**Keywords:** neurosteroids, chronic pain, T-channel (Ca_V_3), T-channel calcium channel blockers, neurosteroid analogs, analgesic (activity)

## Abstract

Since the discovery of the nervous system’s ability to produce steroid hormones, numerous studies have demonstrated their importance in modulating neuronal excitability. These central effects are mostly mediated through different ligand-gated receptor systems such as GABA_A_ and NMDA, as well as voltage-dependent Ca^2+^ or K^+^ channels. Because these targets are also implicated in transmission of sensory information, it is not surprising that numerous studies have shown the analgesic properties of neurosteroids in various pain models. Physiological (nociceptive) pain has protective value for an organism by promoting survival in life-threatening conditions. However, more prolonged pain that results from dysfunction of nerves (neuropathic pain), and persists even after tissue injury has resolved, is one of the main reasons that patients seek medical attention. This review will focus mostly on the analgesic perspective of neurosteroids and their synthetic 5α and 5β analogs in nociceptive and neuropathic pain conditions.

## Introduction

Since the discovery of steroid hormone synthesis in the rat nervous system (Corpechot et al., 1981), numerous studies have shown the pivotal role of steroid hormones in various neuronal functions, such as cognition, memory, affective disorders, neuroprotection, and myelination (Schumacher et al., 2012, 2014; Brinton, 2013; Giatti et al., 2015). These molecules are called neurosteroids, because they are produced in the nervous system by neurons and/or glial cells (Baulieu and Robel, 1990). Today, all steroid hormones that exert an effect on inhibitory and excitatory neurotransmission, regardless of their mechanism(s) and source (whether they are synthetic or endogenously produced), are considered neuroactive steroids (Paul and Purdy, 1992).

Neurosteroids are capable of modulating cell function on different levels. Conventionally, their effects are attributable to specific nuclear hormone receptors (e.g., progesterone) that regulate RNA expression. The onset of such effects is much slower, but the consequential changes may be long lasting. On the other hand, neurosteroids also exert their effects in the nervous system through modulation of various receptor systems and ionic channels (Baulieu et al., 1999; Mensah-Nyagan et al., 1999; Mellon and Griffin, 2002; Belelli and Lambert, 2005). Some of them, such as GABA_A_ and NMDA receptors and/or voltage-dependent T-type Ca^2+^ or voltage-dependent K^+^ channels, are heavily implicated in sensory pathways responsible for mediating anesthesia and analgesia. The present review focuses on the role of neurosteroids in pain pathways, their potential as analgesics in different pain models, and future therapeutic perspectives.

## The Role of Neurosteroids in Pain Pathways

The International Association for the Study of Pain (IASP) defines pain as “an unpleasant sensory and emotional experience associated with actual or potential tissue damage, or described in terms of such damage.” Although acute nociceptive pain has protective value for an organism, promoting survival in life-threatening conditions (Basbaum et al., 2009), prolonged pain that persists even after tissue injury has resolved is one of the main reasons that patients seek medical attention (Costigan et al., 2009).

Some of the first studies with intravenously injected cholesterol showed that steroid molecules could suppress painful information and decrease arousal by exerting an anesthetic-like state in mammals (Cashin and Moravek, 1927). In this study, abdominal surgery on cats was performed after intravenous (IV) administration of cholesterol, and animals were fully recovered. The pregnane class of steroids has become particularly important because of their allosteric modulation of neuronal GABA_A_ receptors. Brot et al. (1997) showed that allopregnanolone mediates its anxiolytic effect by stimulating chloride flux through the channel of GABA_A_ receptors via either binding sites different from the one for benzodiazepines, or *via* sites allosterically linked to the picrotoxin binding site. More recent studies have shown that neurosteroids are influencing the kinetics of synaptic GABA_A_-gated ion channels by prolonging the decay time of phasic responses, and therefore enhancing neuronal inhibition (Belelli and Lambert, 2005; Herd et al., 2007). Furthermore, neurosteroids also exert their effects via extrasynaptic GABA_A_ receptors containing the δ-subunit, thus enabling tonic inhibition (Lambert et al., 2001). This effect was confirmed in a study of Stell et al. (2003) where tonic conductance was significantly reduced in mice lacking the δ-subunit of GABA_A_ receptors, and was not influenced by 5α-pregnan-3α,21-diol-20-one (3α,5α-tetrahydrodeoxycorticosterone,3α,5α-THDOC), a potent stereoselective positive allosteric modulator of the GABA_A_ receptor. The anxiolytic and anesthetic effects of 3α,5α-THDOC were attenuated in these mice (Mihalek et al., 1999). Altogether, these findings could explain the ability of neurosteroids to induce sedation and anesthesia in rodents.

Numerous clinical and animal studies have found that sex hormones can differentially regulate pain perception. For example, testosterone exerts analgesic effect in both humans and animal models, while estrogen can act both as an analgesic and hyperalgesic (Aloisi, 2003; Ceccarelli et al., 2003; Aloisi et al., 2004; Arendt-Nielsen et al., 2004; Aloisi and Bonifazi, 2006). It is well known that fluctuations of estrogen and progesterone during the estrous cycle can influence pain perception and pain threshold (Frye et al., 1992, 1993; Riley et al., 1998; Kuba and Quinones-Jenab, 2005). Furthermore, it seems that estrogen and progesterone may regulate the antinociceptive conformation of mu and kappa opioid receptor heterodimers (Chakrabarti et al., 2010), which makes them immensely important in pain regulation.

**FIGURE 1:**
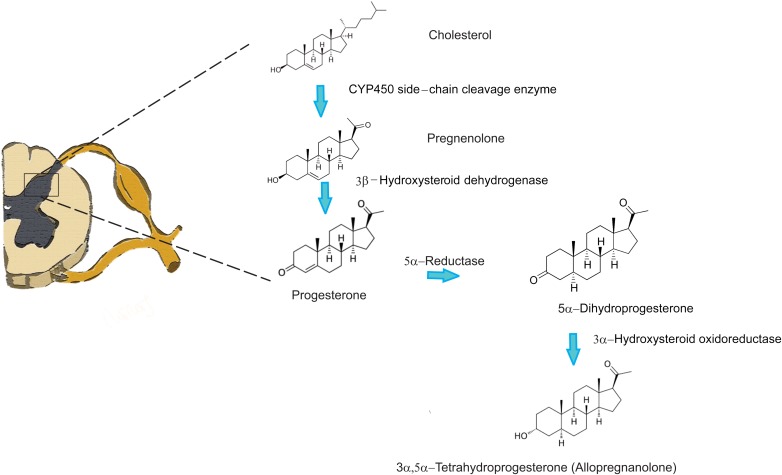
Neurosteroid synthesis in the spinal cord from cholesterol.

Several studies have confirmed the existence of particular enzymes involved in steroidogenesis throughout the central nervous system (CNS) and peripheral nervous system (PNS) (for review Baulieu and Robel, 1990; Compagnone and Mellon, 2000; Mensah-Nyagan et al., 2009). These compounds are crucial for plasticity of the nervous system (Patte-Mensah et al., 2006; Melcangi et al., 2008); therefore, they also play a very important role in pain perception and pain modulation. Interestingly, extensive studies on the dorsal horn region of the spinal cord have not been conducted. However, it is well known that the dorsal horn of the spinal cord plays a pivotal role in the transmission of painful stimuli from the peripheral nociceptors to supraspinal structures. It is also well established that primary afferent fibers coming from peripheral nociceptive neurons, whose cell bodies lie in the dorsal root ganglia (DRG), form synapses with the projection neurons of the dorsal horn of the spinal cord. These neurons further convey information to the brainstem, thalamic, and cortical structures (Millan, 1999, 2002) that are able to modulate nociceptive transmission *via* several descending pathways at the level of the spinal cord. Because modulation of pain perception occurs at the level of dorsal horn neurons in the spinal cord, it is not surprising that a particular set of enzymes, including a CYP450, involved in steroidogenesis was identified (Patte-Mensah et al., 2004; Mensah-Nyagan et al., 2008). An immunohistochemical study by Patte-Mensah et al. (2003) has confirmed that the highest density of these enzymes was detected in superficial layers of laminae I and II, where the first synapses between the nociceptive peripheral sensory neurons and projection neurons are located. Furthermore, homogenates of the rat spinal cord were capable of converting cholesterol into progesterone confirming that the enzymes are indeed functional. Locally synthetized progesterone in the spinal cord has also been shown to stimulate intrinsic spinal anti-nociceptive system *via* kappa and delta opioid receptors and promoting the increase of endorphins *in situ* (Dawson-Basoa and Gintzler, 1997, 1998). Additionally, there is evidence of direct inhibition of allopregnanolone synthesis in the dorsal horn with substance P, a potent pronociceptive neuropeptide (Patte-Mensah et al., 2005). This finding indicates that the presence of neuroactive steroids at the level of the dorsal horn could regulate GABA inhibitory tone, and that the pronociceptive effect of substance P released from primary afferents could be due to the reduction of GABA_A_ receptor activity by downregulating the production of 3α, 5α-THP (allopregnanolone). These data strongly suggest that there is an active process of neurosteroid synthesis in the dorsal horn, particularly progesterone and allopregnanolone (two very potent analgesic neurosteroids) by direct enzymatic conversion (**Figure 1**).

## Endogenous Neurosteroids and Pain

Recent studies suggest that progesterone and its derivatives, dihydroprogesterone (DHP) and 3α, 5α-tetrahydroprogesterone (THP) or allopregnanolone, have a specific neuroprotective action in the central and PNS (Guennoun et al., 2008, 2015; Melcangi et al., 2014; Schumacher et al., 2014). For example, these molecules are capable of exerting beneficial effects in pathological conditions such as traumatic brain injury and spinal cord injury by promoting myelination and preserving white matter. In addition, endogenous neurosteroids show positive therapeutic effect in conditions such as Alzheimer’s and Parkinson’s disease (Irwin et al., 2014; Schumacher et al., 2014). Allopregnanolone, in particular, has been shown to promote neurogenesis by increasing neural progenitor proliferation of subgranular zone of dentate gyrus in adult 3xTgAD mice. Furthermore, in the same mouse model of Alzheimer’s disease, allopregnanolone reversed the conditioned response/associative learning deficits of 3xTgAD mice to a level comparable to non-Tg mice (Wang et al., 2010). Furthermore, in allopregnanolone treated 3xTgAD mice, a significant reduction of β-amyloid production was noticeable (Chen et al., 2011). Allopregnanolone increased dopamine release from nucleus accumbens in freely moving rats (Rougé-Pont et al., 2002) and improved motor performance by promoting neurogenesis of tyrosine hydroxylase immunoreactive neurons is substantia nigra (Adeosun et al., 2012) in the mouse model of Parkinson’s disease.

Numerous studies have also shown that steroids modulate pain sensitivity, either through intracellular/nuclear targets, or by modulation of synaptic transmission, both directly and indirectly *via* second-messenger systems. Potentiation of inhibitory GABA-ergic transmission in the pain pathway is one of the important mechanisms to diminish pain transmission (Zeilhofer et al., 2013). Predictably, analgesic potential of neuroactive steroids that promote GABA-mediated transmission in different pain paradigms is very well established.

Several other mechanisms are involved in analgesia induced by neurosteroids. It has been shown that progesterone, applied subcutaneously, successfully alleviated both mechanical and thermal allodynia in the sciatic nerve injury model, by preventing injury-induced increase of NR-1 subunit of NMDA receptors, as well as expression of PKCγ (Coronel et al., 2011). This reduction of PKCγ prevents the phosphorylation of NR1 subunit of NMDA receptors, which is crucial for receptor facilitation and contributes to the development of central sensitization. Antiallodynic effect of progesterone was also achieved in the spinal cord injury pain model (Coronel et al., 2014). The authors have shown that application of progesterone significantly reduced spinal expression of COX-2 and iNOS after spinal cord injury, revealing an anti-inflammatory activity of this endogenous neurosteroid that could contribute to its analgesic properties. Furthermore, the existence of receptors in dorsal horn neurons, sensitive to progesterone, supports the notion of the importance of neurosteroid induced modulation of pain processing (Labombarda et al., 2010). A particular type of receptor, sigma-1 receptor, a chaperone residing in endoplasmic reticulum, has recently been investigated as a potential target for progesterone binding in the spinal cord (Maurice et al., 2006; Monnet and Maurice, 2006; de la Puente et al., 2009). Sigma-1 receptors chaperon proper folding of nascent proteins. However, in certain conditions, such as binding of agonists, they could translocate to the cell membrane and interact with different G-protein coupled receptors and ionic channels. Previous studies have confirmed the presence of sigma-1 receptor in the dorsal horn neurons (de la Puente et al., 2009), as well as on DRG (Mavlyutov et al., 2016) which implicates them in the development of neuropathic pain and spinal sensitization after nerve injury. It has also been shown that activation of sigma-1 receptors leads to increased activity of PKC and PKA, which in turn induces phosphorylation of NR1 subunit of NMDA receptors, a process highly implicated in the development of central sensitization (Kim et al., 2008). Ortíz-Rentería et al. (2018) discovered that blocking sigma-1 receptors by progesterone leads to a significant decrease of TRPV1-dependent pain produced by capsaicin. Different authors have shown that treatment of sciatic nerve constriction and orofacial pain models with progesterone also successfully reduced mechanical and thermal allodynia (Dableh and Henry, 2011; Kim et al., 2012).

Of particular interest is dehydroepiandrosterone (DHEA), one of the first discovered neurosteroids (Baulieu et al., 1999). DHEA is found to be converted from pregnenolone by cytochrome P450c17 in the CNS. Although it can be found in human plasma, in rodents, plasma levels are extremely low, most likely since cytochrome P450c17 does not exist in rodent adrenals. However, DHEA synthesis exists in the spinal cord (Kibaly et al., 2005), a pivotal part of the sensory pathways, important for nociceptive transmission. Therefore, the importance of DHEA in modulation of pain perception should be considered. Kibaly et al. (2007) have found that when injected either peripherally (subcutaneous) or centrally (intrathecal), DHEA exerts pronociceptive effects by reducing the pain thresholds to painful stimuli. However, the repeated injections of DHEA exert a sustained analgesic effect. This indicates that DHEA exerts its acute algogenic effects most likely via either direct allosteric modulation of NMDA or P2X receptors, or via sigma-1 receptors, which in turn enhances phosphorylation of NR1 subunit of NMDA receptors (Yoon et al., 2010), leading to increased sensitization of pain pathways. On the other hand, delayed analgesic effect of DHEA could be explained by its metabolism to androgens in the spinal cord, such as testosterone, that exert analgesic effects (Kibaly et al., 2007).

Dehydroepiandrosterone seems to possess another feature, related to neuroprotection. A recent study of Lazaridis et al. (2011) indicated that DHEA prevented neuronal apoptosis by interacting with transmembrane tyrosine kinase receptor TrkA. TrkA receptors are a known group of target receptors for the nerve growth factor (NGF). Via these receptors, NGF prevents cell apoptosis. Thus, the authors have discovered that, by binding to TrkA, DHEA exerts an antiapoptotic effect in HEK-293 cells, and reversal of apoptotic loss of TrkA positive sensory neurons in DRG of NGF null mouse embryos.

On the other hand, NGF/TrkA signaling pathway has already been implicated in pain transmission (Hirose et al., 2016). From the developmental standpoint, NGF is necessary during fetal period for normal growth of sensory nerve fibers belonging to pain pathways. However, during the adulthood, an NGF increase occurs during peripheral inflammation and nerve injury. This increase of peripheral NGF leads to the sensitization of surrounding nerves inducing pain, which is a protective response to prevent further tissue injury. But, if the inflammation and nociception persist, the protective value of increased NGF is lost leading to central and peripheral sensitization of pain pathways, and to the development of chronic pain states. This notion has been confirmed in the study by Ashraf et al. (2016) where an experimental compound has been utilized to antagonize effects of NGF via blocking the TrkA receptors, and therefore reducing pain and joint damage in rat models of inflammatory arthritis. Interestingly, a synthetic analog of DHEA, named BNN27, has been recently shown to interact with TrkA receptors (Pediaditakis et al., 2016) on DRG neurons without exerting pronociceptive behavior. This indicates a selective action against neurodegeneration but not influencing the pain pathways. Therefore, the rogue neurosteroid DHEA, and synthetic analogs, could have a more pleiotropic role, by simultaneously interacting with different receptor systems and hence, exerting different effects, that could be both beneficial and detrimental, depending on the activated pathways, age, and pathologies. Further studies are needed to elucidate the mechanisms of these dichotomous effects of DHEA in the pain pathway.

Progesterone’s metabolites, DHP and THP, have also been proven to act as analgesics in various pain models. For example, in the sciatic nerve crush injury model, progesterone and DHP successfully alleviated thermal nociception. This is possibly accomplished by restoring the thickness of the myelin and reducing the density of the fibers, as well as normalizing the function of Na^+^/K^+^-ATPase pump activity (Roglio et al., 2008). In chemically induced neuropathies, such as streptozotocin-induced diabetic neuropathy and chemotherapy-induced neuropathy, progesterone, allopregnanolone, and 5α-DHP have alleviated either thermal and/or mechanical nociception (Leonelli et al., 2007; Meyer et al., 2010, 2011).

Pathirathna et al. (2005b) have shown that allopregnanolone successfully alleviated mechanical and thermal hyperalgesia in the neuropathic pain model of loose sciatic nerve ligation in rats by modulating both T-type Ca^2+^ channels (T-channels) and GABA_A_ receptors. Specifically, allopregnanolone was a more potent analgesic than its analogs, which only inhibited T-channels or potentiated GABA_A_-gated currents.

Additionally, Ayoola et al. (2014) have found that local paw injections of epipregnanolone, an endogenous 5β-reduced neurosteroid without potentiating effects at GABA_A_ receptors, successfully alleviated mechanical and thermal sensitivity in both wild-type mice and rats, but not in Ca_V_3.2 T-type calcium channel knock-out mice. This suggests that the antinociceptive effect of epipregnanolone is mediated largely by inhibition of T-channels in peripheral nociceptors.

**FIGURE 2:**
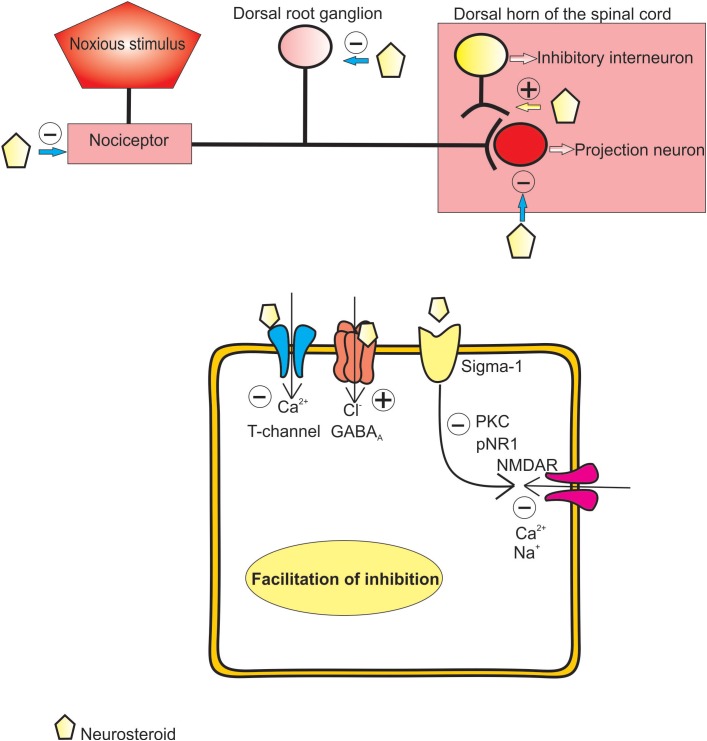
Proposed mechanisms of analgesic effect of endogenous and synthetic neurosteroids.

Taken together, these data strongly suggest that endogenously produced neuroactive steroids are very potent analgesics in different pain models, and that they exert their analgesic effects *via* various receptor systems and ion channels, most notably GABA_A_ receptors and T-type calcium channels (**Figure 2**). On one hand, their ability to evoke effects through different receptor systems, either directly or through second-messenger systems, makes them an excellent alternative to conventional therapeutic options for treating various pain states. On the other hand, drugs that target so many receptor systems often produce various adverse events in humans. Therefore, creating a potent, but more selective, neuroactive steroid would be a focus of future studies of these interesting compounds.

## Analgesic Properties of 5α- and 5β-Reduced Steroid Analogs

The role of GABA_A_ receptors as the main inhibitory receptors in pain pathways is well established (Millan, 1999); however, the role of voltage-gated Ca^2+^ channels (VGCCs) has not been explored as thoroughly. Based upon the membrane potential that activates them, VGCCs can be divided into two categories: high-voltage-activated (HVA) and low-voltage-activated T-type Ca^2+^ channels. Of interest for this review are T-type Ca^2+^ channels that are important targets for many analgesic neurosteroids. Since the discovery of the role of these channels in neuronal excitability, and their presence on peripheral sensory neurons whose cell bodies are located in the DRG (Carbone and Lux, 1984), a growing interest for studying these channels in pain transmission has arisen (Todorovic et al., 2001; Bourinet et al., 2005; Jacus et al., 2012; Rose et al., 2013). Therefore, synthetic analogs of neuroactive steroids with affinity for T-channels have been synthetized to investigate their role in both acute and chronic pain models.

Among several synthetic 5α-analogs tested, our previous work has shown that ECN, [(3β,5α,17β)-17-hydroxyestrane-3-carbonitrile], a potent enantioselective blocker of T-channels without potentiating effects at GABA_A_ receptors (Todorovic et al., 1998), induced potent analgesia when applied locally as an intraplantar injection in healthy rats (Pathirathna et al., 2005a). Furthermore, when combined with CDNC24, a GABA_A_ selective neurosteroid without analgesic effect *per se*, the antinociceptive effect of ECN was greatly potentiated. This synergistic analgesia was abolished with a GABA_A_-receptor antagonist biccuculine, indicating that there is an interplay between GABA_A_ receptors and T-channels in peripheral nociceptors that helps them to work in concert when alleviating acute pain (Pathirathna et al., 2005a).

These two 5α-reduced analogs have been shown to alleviate pain in chronic neuropathy as well. Our group has shown that in a model of chronic constrictive injury (CCI) of the sciatic nerve, local intraplantar injections of either ECN or CDNC24 more selectively alleviated thermal nociception in neuropathic animals than in a sham group, as compared to allopregnanolone or alphaxalone (Pathirathna et al., 2005b). This can be explained by the fact that synthetic neurosteroids are more selective to either GABA_A_ and/or T-channels, while allopregnanolone and alphaxalone have many other targets on which to exert their antinociceptive effect.

The fact that CDNC24 exerted effect in injured but not in healthy animals can be explained by the changes in expression and/or conductance of GABA_A_ receptors during injury. The study of Xiao et al. (2002) has confirmed the increase in mRNA levels for α_5_ subunit of GABA_A_ receptors on the cell bodies of peripheral sensory neurons, while Yang et al. (2004) have shown the upregulation of α_5_ subunit of the GABA_A_ receptor in the spinal cord after nerve injury. It is noteworthy that both alphaxalone and allopregnanolone may exert antinociceptive effect through potentiating GABA_A_ currents as well as inhibiting T-currents. More importantly, after blocking the GABA-ergic effect with biccuculine, a potent analgesia could still be observed, indicating that a great portion of neurosteroid-induced analgesic effect is related to the T-channel inhibition. As previously mentioned, neuroactive steroids have various targets to prevent mechanisms that trigger plasticity changes in the nervous system. Perhaps a future strategy of preventing neuropathic pain could be achieved by blocking T-channels heavily involved in neuronal excitability. Furthermore, systemic intraperitoneal administration of ECN effectively reversed mechanical and thermal hyperalgesia in painful diabetic neuropathy in morbidly obese leptin-deficient ob/ob mice (Latham et al., 2009). In addition, some studies have implicated GABA_A_ receptors in the dorsal horn of spinal cord as important targets for treatment of painful diabetic neuropathy (Jolivalt et al., 2008). These data strongly suggest that neurosteroids targeting either T-type channels and/or GABA_A_ receptors may be beneficial in treatment of intractable pain associated with peripheral diabetic neuropathy.

Recent behavioral and immunohistological studies have confirmed the presence of both GABA_A_ receptors and the Ca_V_3.2 isoform of T-channels on the peripheral nociceptors (Rose et al., 2013; Obradovic et al., 2015). Ca_V_3.2 isoform of T-channels has also been found presynaptically in the spinal cord where these channels support glutamate release from the central endings of nociceptive sensory neurons (Jacus et al., 2012). In addition, the Ca_V_3.1 isoform of T-channels controls the opioidergic descending inhibition from the low-threshold spiking GABAergic neurons in the periaqueductal gray (PAG; Park et al., 2010). Some previous studies showed that an increase of intracellular calcium in Purkinje cells increases sensitivity to GABA (Llano et al., 1991). On the other hand, intracellular calcium decreases the affinity of GABA receptors in sensory neurons of the bullfrog (Inoue et al., 1986). Perhaps, in the PNS, blocking the T-channels leads to the decreased intracellular Ca^2+^, which in turn leads to the increased activity of GABAergic inhibition. Overall, these data suggest that there is a strong interaction between the GABAergic inhibitory system and T-type channels in both central and peripheral components of the pain pathway. However, exact mechanisms of this interaction remain to be determined.

Previous work from our lab has also shown that synthetic 5β-reduced neurosteroids can successfully alleviate somatic pain (Todorovic et al., 2004). The steroid structures tested both *in vitro* and *in vivo* contain either 3-cyano and 17-hydroxyl groups or 3-hydroxyl and 17-cyano groups. These selective T-channel blockers have exerted significant and dose-dependent analgesia when injected locally into the plantar surface of the hind paw in healthy rats. Specifically, (3β,5β,17β)-3-hydroxyandrostane-17-carbonitrile (3β-OH) was one of the most effective synthetic 5β- reduced neurosteroids in alleviating thermal nociception. Interestingly, the potency to block isolated T-currents in DRG neurons *in vitro* corresponded well to their potency to exert thermal antinociception *in vivo*. Furthermore, 3β-OH has been recently shown to possess hypnotic effect and was able to induce loss of righting reflex in neonatal rats without causing harmful effects to the brain of exposed animals (Atluri et al., 2018). It is reasonable to assume that both analgesic and hypnotic effects were likely exerted by blocking low voltage activated T-channels, suggesting that this novel synthetic neurosteroid with a specific and selective mechanism of action could be used for preemptive analgesia and anesthesia. However, further studies are needed to test this notion, and to investigate the effects of other synthetic neurosteroid analogs in both acute and chronic pain models.

## Conclusion

Endogenous neurosteroids are very potent molecules with effects on many crucial processes in the nervous system. By targeting several different receptor systems, they are able to reduce maladaptive changes in the sensory nervous system, which in turn could prevent the development of central sensitization and chronic pain states. Perhaps, increasing the production of endogenous neurosteroids, such as progesterone and allopregnanolone, in the spinal cord and peripheral nerves would be a future therapeutic option for treating various pain states in humans. This strategy has already been employed in several animal studies and has proven successful in neuropathic, inflammatory, as well as in postoperative pain models (Aouad et al., 2009; Giatti et al., 2009; Hernstadt et al., 2009; Mitro et al., 2012; Xiong et al., 2017). By targeting specific translocator protein-18 kDa (TSPO) and/or liver X receptors (LXR), it is possible to increase neuronal steroidogenesis, thus preventing systemic endocrine effects that could appear as a consequence of systemic application of neurosteroids.

On the other hand, synthetic 5α- and 5β-reduced steroid analogs have great potential in treating acute and chronic pain. Since rigid steroid molecules can be sculpted to generate more selective compounds towards GABA_A_ receptors and T-channels, they may be the most interesting in terms of further development as novel pain therapies. An example would be 3β-OH, a synthetic neurosteroid with hypnotic and analgesic properties, that could be a promising new agent to reduce postsurgical hyperalgesia, when applied as a part of a balanced anesthesia, thus potentially reducing the necessity for other analgesic drugs, such as opioids after surgery. By specifically targeting key ion channels that contribute to the modulation of pain perception, synthetic neurosteroids could in turn alleviate pain in patients, hopefully with less adverse events than currently used therapies. Future clinical trials are necessary to investigate their analgesic potential and safety profile in the human population.

## Author Contributions

SJ wrote the main draft of the manuscript. ST, VJ-T, and DC revised the draft of the manuscript and made final corrections. All authors approved the final version of the manuscript.

## Conflict of Interest Statement

The authors declare that the research was conducted in the absence of any commercial or financial relationships that could be construed as a potential conflict of interest.
